# Chorioretinal abnormalities in idiopathic intracranial hypertension: case reports

**DOI:** 10.1186/s40942-022-00403-2

**Published:** 2022-07-22

**Authors:** Leonardo E. Ariello, Luiz Guilherme Marchesi Mello, Sérgio Luis Gianotti Pimentel, Mário L. R. Monteiro

**Affiliations:** 1 Division of Ophthalmology, University of São Paulo Medical School (USP), Av. Dr Enéas de Carvalho Aguiar, 255, Cerqueira César, São Paulo, SP 05403-001 Brazil; 2 Departamento de Medicina Especializada, Federal University of Espírito Santo, Espírito Santo, Brazil

**Keywords:** Choroidal neovascularization, Idiopathic intracranial hypertension, Papilledema, Choroid, Choroidal folds, Retinal neovascularization

## Abstract

**Background:**

Papilledema is the main ocular finding in patients with idiopathic intracranial hypertension (IIH) although several chorioretinal abnormalities may also occur and contribute to visual loss. The purpose of this paper is to describe two cases of chorioretinal abnormalities associated with idiopathic intracranial hypertension: one with choroidal folds and another with polypoidal choroidal vasculopathy, an extremely unusual ocular complication in the disease.

**Case presentation:**

Case 1: A 47-year-old woman previous diagnosed with idiopathic intracranial hypertension treated with weight loss and acetazolamide that over the following 6 months had optic disc edema gradually resolved. The patient was follow-up for a period of 10 years and the papilledema disappeared, but choroidal folds remained unchanged. Case 2: A 61-year-old female patient was seen as a follow-up examination of a 5-year history of IIH that presented with papilledema. The patient was asymptomatic but fundoscopy evaluation revealed a yellowish white peripapillary subretinal nodular lesion temporally in OD. Multimodal imaging studies were made, and the patient was diagnosed with a rare and just recent described association of IIH and polypoidal choroidal vasculopathy.

**Conclusion:**

Papilledema, RNFL and retinal ganglion cell loss are the most common structural complications of IIH, but chorioretinal complications are important findings and should be carefully evaluated in such patients. Awareness of such occurrence and the use of appropriated clinical and multimodal imaging studies are of great importance for its early detection, leading to proper treatment and prevention of further visual loss.

## Introduction

Idiopathic intracranial hypertension (IIH) is characterized by signs and symptoms of increased intracranial pressure of unknown etiology. Headache, transient visual obscuration, pulsatile tinnitus, and neck pain are the most typical symptoms although uncommon presentations can also occur [1, 2]. The disease is strongly related to obesity, especially in middle-aged women, but other endocrine and metabolic disorders may also be associated [1].

The diagnosis of IIH is based on the modified Dandy criteria and, in addition to clinical symptoms, include: (i) an elevated lumbar puncture opening pressure (> 25 cmH_2_O in lateral decubitus) with normal cerebral spinal fluid (CSF) composition; (ii) normal brain parenchyma without hydrocephalus, mass or structural lesion and no meningeal enhancement or venous sinus thrombosis seen on neuroimaging; (iii) clinical evidence of papilledema with normal neurological examination except for sixth nerve palsy [3].

While visual function is usually preserved in the early stages of the disease, the persistence of papilledema particularly in chronic cases may lead to a progressive loss of retinal nerve fiber layer (RNFL) and retinal ganglion cells. Consequently, chronic IIH can produce an array of visual field (VF) abnormalities, including blind spot enlargement, inferior nasal depression, and generalized VF constriction [4]. Therefore, early diagnosis, prompt treatment, and management of complications are essential to avoid severe and permanent visual loss [5].

In addition to papilledema and retinal neural loss, IIH may also be associated with several secondary retinal lesions, such as macular exudates, retinal hemorrhages, subretinal neovascular membrane, central retinal vein occlusion, and retinal folds [6]. Choroidal abnormalities also occurs and ranging from a commonly found choroidal folds to an unusual, recently described, polypoidal choroidal vasculopathy (PCV) [7, 8]. When such lesions are present, a significant diagnostic confusion could pave the way for an erroneous treatment with potential risk to visual prognosis.

The purpose of the present paper is to describe two cases of choroidal abnormalities associated with IIH and to review chorioretinal findings associated with this disease. We also first report a multimodal imaging analysis of PCV in a patient with IIH, including the use of optical coherence tomography angiography (OCT-A).

## Case report

### Case 1

A 47-year-old woman complained over the last 3 months of occasional flashes and glare in the temporal side of both eyes (OU), lasting for a few seconds. Past medical history was significant only for obesity (weight 106 kg, height 1.60 m) and hyperopia OU. On examination, best-corrected visual acuity (VA) was 20/25 in the right eye (OD, with +5.50 spherical diopters) and 20/25 in the left eye (OS, with +4.75 spherical diopters). Pupillary examination, extraocular movements, and slit lamp examination were unremarkable. Fundoscopy revealed a marked papilledema and choroidal folds extending from the optic disc to the macula in OU. Standard automated perimetry revealed an enlarged blind spot and nasal sensitivity loss OU. Magnetic resonance imaging (MRI) and magnetic resonance venography (MRV) of the brain only demonstrated an empty sella turcica. CSF opening pressure revealed raised intracranial pressure (28 cmH_2_O) with normal biochemical and cytological analysis.

**Fig.1:**
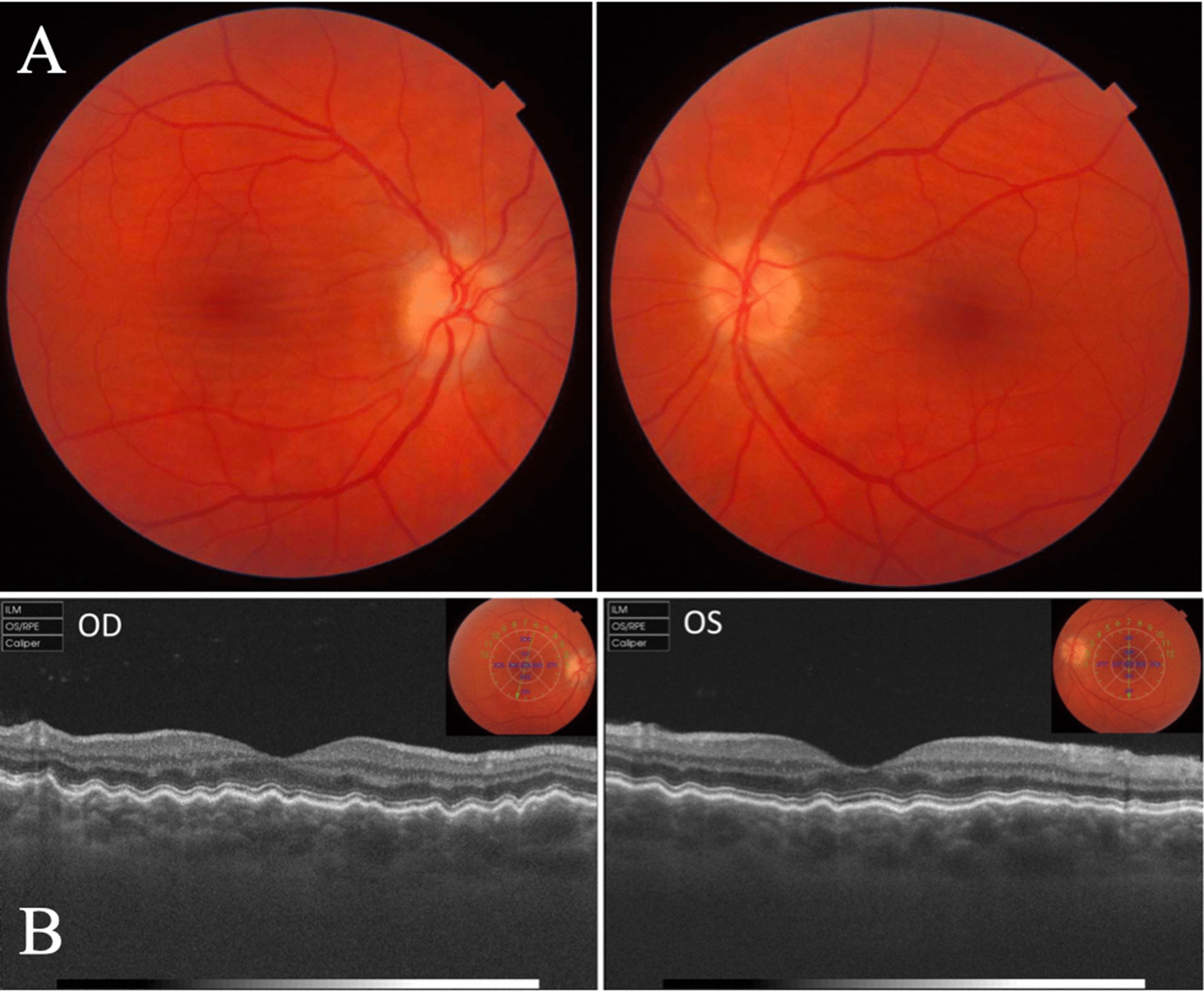
Right and left eye color fundus photographs showing a slightly pale grayish optic disc and horizontal chorioretinal folds in the macular area in both eyes **A**. OCT showing chorioretinal folds and wrinkles in both eyes **B**

A diagnosis of IIH was established and oral acetazolamide was started associated with dieting to achieve weight loss. Over the following 6 months optic disc edema gradually resolved and treatment was maintained for 3 years. Papilledema disappeared but choroidal folds remained unchanged in a follow-up period of 10 years (Fig. 1A), despite normalization of intracranial pressure. Optical coherence tomography (OCT) revealed a marked wrinkling of the choroid in OU (Fig. 1B). Subfoveal choroidal thickness (CT) was 309 µm in OD and 318 µm in OS. VA and VF remained unchanged during the follow-up period.

### Case 2

A 61-year-old female patient was seen as a follow-up examination of a 5-year history of IIH that presented with papilledema, elevated intracranial pressure (35 cmH_2_O) with normal CSF composition, normal MRI and MRV. At the diagnosis, she denied visual or systemic complains. Past medical history was significant for systemic arterial hypertension and grade II obesity at presentation. Treatment was accomplished with significant weight loss and oral acetazolamide leading to complete resolution of papilledema over the following 2 years.

**Fig.2:**
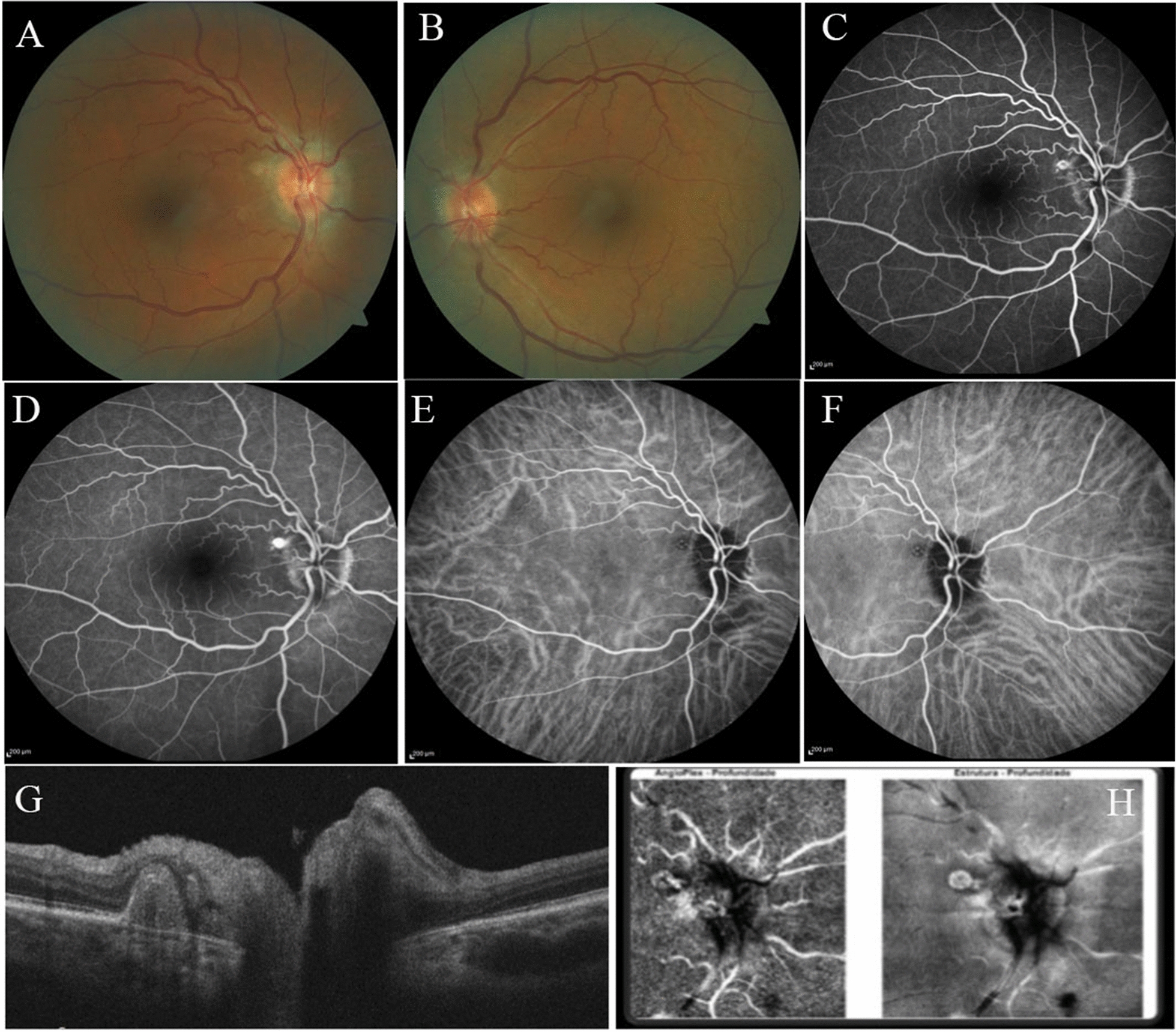
Right **A** and left eye **B** color fundus photographs showing a slightly pale grayish optic disc with peripapillary atrophy of the retinal pigment epithelium in both eyes and a yellowish-white peripapillary subretinal nodular lesion temporally in right eye. **B**. Right eye fluorescein angiography showing early **C** and late peripapillary nodular hyperfluorescence **D**. Right eye early phase indocyanine green angiography showing polypoidal lesions surrounded by hypofluorescent halo **E**, **F**. Cross-sectional optical coherence tomography scan along the peripapillary nodule revealing dome shaped RPE elevation with moderate internal reflectivity **G**. En face optical coherence tomography angiography showing RPE elevation **H**

On examination, VA was 20/20 in OU, with normal pupillary examination, extraocular movements, intraocular pressure, and anterior segment biomicroscopy. Fundoscopy revealed a slightly pale grayish optic disc with peripapillary atrophy of the retinal pigment epithelium (RPE) in OU and a yellowish white peripapillary subretinal nodular lesion temporally in OD (Fig. 2A, B). Fluorescein angiography showed a peripapillary nodular hyperfluorescence (Fig. 2C, D) and early phase indocyanine green angiography (ICGA) disclosed polypoidal lesions surrounded by hypofluorescent halo in OD (Fig. 2E, F). Cross-sectional OCT scan of the peripapillary lesion revealed a dome shaped RPE detachment (PED) with moderate internal reflectivity and pachychoroid (Fig. 2G). Subfoveal CT measurements were 370 µm in OD and 311 µm in OS. *En face* OCT-A demonstrated RPE elevation (Fig. 2H). VF examination demonstrated an enlarged blind spot in OU. Imaging studies were unremarkable in the OS and CSF opening pressure was 18 cmH_2_O.

Neurologic examination and extensive laboratory investigation were normal. A diagnosis of a peripapillary PCV associated with IIH was made. A closer follow up was adopted without further treatment since no signs of active PCV or IIH were detected.

## Discussion

Retinal lesions are important causes of vision loss in IIH. Subretinal neovascular membrane with subretinal fluid and hemorrhage are the most common chorioretinal complication in IIH associated with visual loss [6, 9]. Macular exudates and superficial retinal hemorrhages are also frequently described but rarely affect patient vision [10]. The pathogenesis of subretinal neovascular membrane in IIH is not clearly understood, but it is believed that axoplasmic flow stasis and angiogenic stimulus secondary to choroidal hypoxia plays an important role in its pathophysiology [11, 12].

The cases here presented exemplify one common and another extremely unusual choroidal abnormality that may occur in patients with IIH. Choroidal folds, as shown in our first case, is the most common choroidal manifestation associated with papilledema in IIH and frequently persists after resolution of papilledema and normalization of the intracranial pressure [13], as observed in our case 1. Indeed, they are not exclusive of papilledema, and it has been described in a variety of ophthalmic disorders such as orbital infiltrative process, orbital and ocular tumors, ocular hypotony, scleral buckles, among others [14]. Folds are a response that minimize stored elastic energy when a surface is compressed and exceed a critical stress and strain of a tissue [15, 16]. The patter of a fold depends on the loading force and geometry of surrounding structures [17]. In papilledema, chorioretinal folds represent structural consequence of posterior compression of the optic nerve secondary to raised intracranial pressure with consequent anterior displacement of the ocular wall. Besides, it’s also believed that distention of the prelaminar axons due to axoplasmic flow stasis and secondary expansion of the optic nerve head would displace centrifugally the juxtapapillary retina and generates them [18]. In the eye, folds are represented in three major patterns; i) peripapillary wrinkles, ii) retinal folds, and iii) choroidal folds, the latter usually associated with higher levels of intracranial pressure and less common than the others. [7, 19]

It is also important to consider that choroidal abnormalities in a scenario of raised intracranial pressure may not only involves structural changes, exemplified as wrinkles and folds. Angiogenic stimulus in a hypoxically choroidal environment are reported in chronic IIH and neovascularization can occur [20]. Peripapillary neovascular membranes is a relatively common manifestation of chronic hypoxic in IIH, but most recently PCV has also been described in association with the disease [8]. Our second case is interesting due to a very rare presentation of a peripapillary PCV in an asymptomatic patient with chronic papilledema. As far as we know, this is the second report of PCV in IIH, and the first with OCT-A imaging analysis. The yellowish-white peripapillary subretinal nodular lesion raised the suspicion of an underlying condition and since peripapillary abnormalities such as chorioretinal folds and neovascular membranes are well described and harbor a risk of visual impairment, a multimodal analysis was made. Interestingly, the patient had increased subfoveal CT in the affected eye revealing a possible propensity for development of PCV, precipitated by the eye wall deformity and the possible hypoxic environment caused by papilledema due to persistently raised intracranial pressure.

PCV is a vascular disease of the choroid first described in 1990 [21]. The polyps seen on fundoscopy as orange nodules represent the aneurismatic dilatation adjacent of a ramified vascular network beneath the RPE. It is characterized by recurrent serosanguineous maculopathy with poor visual prognosis [22]. The gold standard for its diagnosis is still ICGA, demonstrating the polypoidal dilatations emerging from the choroid vasculature but the non-invasive properties and high accuracy for diagnosis also validated OCT as important exam [23]. There has been ongoing debate regarding the pathogenesis of PCV. It is believed to be a subtype of age-related macular degeneration in which an abnormal branching vascular network with an aneurysmal dilatation referred as polyps emerge underneath the RPE [24]. The disease probably represent a chronic process of inner choroid vasculature in which systemic hypertension cause hyalinization of vessel wall, loss of smooth muscles and pericytes, aneurismatic dilatations and exudation [25, 26]. As previous described, chronic posterior globe flattening may displace choroidal structures anteriorly causing folds and wrinkles. It is believed that a mixed mechanism is responsible for the development of PCV in papilledema since structural abnormalities secondary to compression of the optic nerve head and chronic choriocapillaris ischemia may lead to a hypoxic environment, reactive angiogenesis, and formation of polyps [27].

As seen in our second case, detecting chorioretinal complications in IIH depends in part of multimodal analysis. OCT, OCT-A, fluoresceine angiography and ICGA are useful diagnostic modalities that help clinician to detect alterations not seen on retinography and ophthalmic examination. Besides it’s noninvasive nature, OCT and OCT-A has been widely used to access optic neuropathy. RNFL thickness measurement is helpful in quantifying changes in papilledema, even in the milder ones [28]. Ganglion cell complex analysis provides measurements less influenced by axonal changes and optic disk edema and, therefore, is more reliable indicator of retinal neural loss in the presence of papilledema [29]. OCT-A is a technology that allow imaging the retina microvasculature without injection of intravenous dye. It has been used in the evaluation of macular disease, such as neovascular type of age-related macular degeneration, macular telangiectasia, diabetic maculopathy, and others retinal vascular diseases [30]. OCT is also useful detecting polypoidal lesion and choroidal folds. Shallow elevations of the RPE known as the ‘double layer sign’, subretinal pigment detachment ring-like lesion associated with thumb-like PED has high specificity and sensibility for the diagnosis of PVC [31, 32]. For chorioretinal folds, fundoscopy is usually sensitive enough to detect, but FA and OCT may aid information on choroidal folds when peripapillary wrinkles and retinal folds are not present, supporting its diagnosis [33, 34]. Therefore, the use of OCT and OCT-A in IIH is essential not only to evaluate the degree of papilledema and ganglion cell loss, but also for early detection of complications as the development of chorioretinal folds and polypoidal lesions. [35, 36].

## Conclusion

In conclusion, papilledema, RNFL and retinal ganglion cell loss are the most common structural complications of IIH, but chorioretinal complications are important findings and should be carefully evaluated in such patients. Awareness of such occurrence and the use of appropriated clinical and multimodal imaging studies are of great importance for its early detection, leading to proper treatment and prevention of further visual loss.

## Data Availability

The datasets used and analysed during the current study are available from the corresponding author on reasonable request.
